# Curve flexibility assessment on AIS surgical candidates using ultrasonic imaging method - a preliminary study

**DOI:** 10.1186/1748-7161-10-S1-O39

**Published:** 2015-01-19

**Authors:** Edmond Lou, Rui Zheng, Lawrence Le, Doug Hill, Jim Raso, Douglas Hedden, James Mahood, Marc Moreau

**Affiliations:** 1 University of Alberta, Alberta, Canada; 2 Alberta Health Services, Alberta, Canada

## Objective

Bending radiographs are routinely used to determine curve flexibility during planning of scoliosis surgery. However, the cumulative dose of ionizing radiation is a concern, especially in growing children. Ultrasound (US) imaging method has been used to measure the coronal curvatures up to 45° Cobb. The objectives were to investigate the feasibility of using US method to scan AIS surgical candidates, to measure the curve flexibility and to determine its correspondence with the radiographic measures.

## Methods

Four female AIS surgical candidates (age: 14.6±1.6 years) consented to participate. Both radiographs and US images, in standing, left bending and right bending positions, were obtained prior to scoliosis surgery. Routine supine bending radiographs for surgical planning were assessed, whereas the US was acquired in the prone position. The post-op standing radiographs were acquired one week after the surgery. One rater blinded to the clinical information measured the curvature with one week apart between modalities. Eight curves, 4 right thoracic, 1 thoracolumbar and 3 lumbar curves, were measured. The curve correction angles were calculated by subtracting the bending curves from the standing Cobb angles. The mean absolute deviation (MAD), standard deviation (SD) and correlation coefficient (R) of the correction angles between the US images and radiographs were compared.

## Results

The pre-op Cobb angles from standing radiographs and ultrasound images were 53±16° and 49±16°, respectively. The correction angles (MAD±SD) measured from the bending radiographs, bending ultrasound and the post-op radiographs were 31±8°, 39±10°, and 31±9°. The (MAD±SD and R) of the correction angles between the bending radiographs versus the post-op radiographs, and the bending ultrasound versus the post-op radiographs were (9±4°, 0.34) and (8±6°, 0.83) respectively. Although the overall curve correction was similar between the radiography and ultrasound measurements, the ultrasound measurements showed a higher correlation between the predicted correction and the final outcomes.

## Conclusions

Ultrasound imaging method has a potential to measure the coronal curvature of the AIS surgical candidate. Although the curve flexibility can be estimated by the US method, the results showed more correction compared to the bending radiography measurements. This may be partially due to the difference in prone and supine bending posture. The correction measurements from the US and post-op measurements presented higher correlation than the measurements between pre-op and post-op radiographs. However, due to a limited number of participants, a definite conclusion cannot yet be made.

